# Large Retroperitoneal Hematoma: A Rare Intraoperative Complication of Total Vaginal Hysterectomy

**DOI:** 10.7759/cureus.16760

**Published:** 2021-07-30

**Authors:** Anna Fuchs, Kirthik Nathan Parthasarathy, Abigail Coots, Nikunj R Chauhan, Xuezhi Jiang

**Affiliations:** 1 Obstetrics and Gynecology, Drexel University College of Medicine, Philadelphia, USA; 2 Obstetrics and Gynecology, Reading Hospital Tower Health, Reading, USA; 3 Trauma, Acute Care Surgery, and Surgical Critical Care, Reading Hospital Tower Health, Reading, USA; 4 Interventional Radiology, Reading Hospital Tower Health, Reading, USA

**Keywords:** retroperitoneal hematoma, abnormal uterine bleeding, surgical complication, total vaginal hysterectomy, intraoperative hemodynamic instability, gynecologic surgery

## Abstract

Retroperitoneal (RP) hematoma is a rare complication of total vaginal hysterectomy. A 45-year-old female G4P3013 with a history of abnormal uterine bleeding refractory to treatment by endometrial ablation and stress urinary incontinence underwent total vaginal hysterectomy, bilateral salpingectomy, bilateral uterosacral ligament suspension, anterior colporrhaphy, and cystoscopy. After the hysterectomy the left uterine artery pedicle was hemostatic; however, the patient became hemodynamically unstable and anemic. Laparoscopy revealed a stable zone III RP hematoma. Intraoperative observation revealed no further expansion of the hematoma. Left iliac angiography and aortography revealed there was no extravasation from the uterine arteries and gonadal vessels. Four days post-operative abdominal CT showed a stable hematoma. Hemodynamic instability resolved over the post-operative course. RP hematoma must be included in the differential for the evaluation of acute intraoperative hemodynamic instability with an unclear source.

## Introduction

Hemorrhage is an intraoperative complication that occurs in 2.5% of women who undergo vaginal hysterectomy [1]. It usually occurs secondarily to a loose vascular pedicle or visceral injury. Loose vascular pedicles can lead to intra-abdominal and RP hemorrhage. Though there is an awareness that retroperitoneal (RP) hematoma is a possible complication of total vaginal hysterectomy, it has not been reported with frequency in previous literature. There are no statistics available to indicate the rate or the outcome of RP hematoma as a complication of this procedure.

Furthermore, knowledge of RP hematoma as a complication of any gynecological surgery is scarce because there are few case reports and no studies in the literature. There is one literature review by Rafi and Muppala; however, this covers the topic of obstetrical RP hematoma [1]. It is known that RP hematoma can initially be difficult to diagnose and is a cause of significant morbidity and mortality. This case demonstrates that despite the limited information on the frequency and consequence of RP hematoma in the context of gynecology, it is important to keep RP hematoma in the differential and to initiate rapid treatment vaginally, laparoscopically, or by laparotomy to locate and control the source of hemorrhage.

## Case presentation

**Figure 1:**
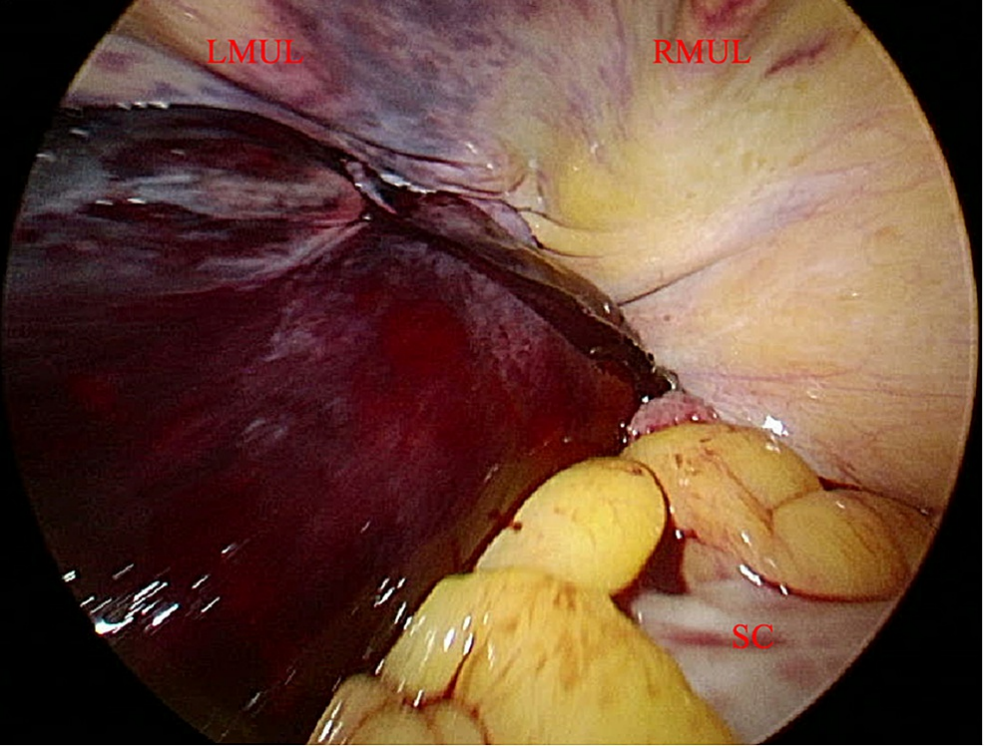
Laparoscopic image of the retroperitoneal hematoma with the camera located in the left upper quadrant at Palmer’s point, directed at the dependent pelvis. Sigmoid colon deflected to the patient’s right from mass effect. LMUL, left median umbilical ligament; RMUL, right median umbilical ligament; SC, sigmoid colon

**Figure 2:**
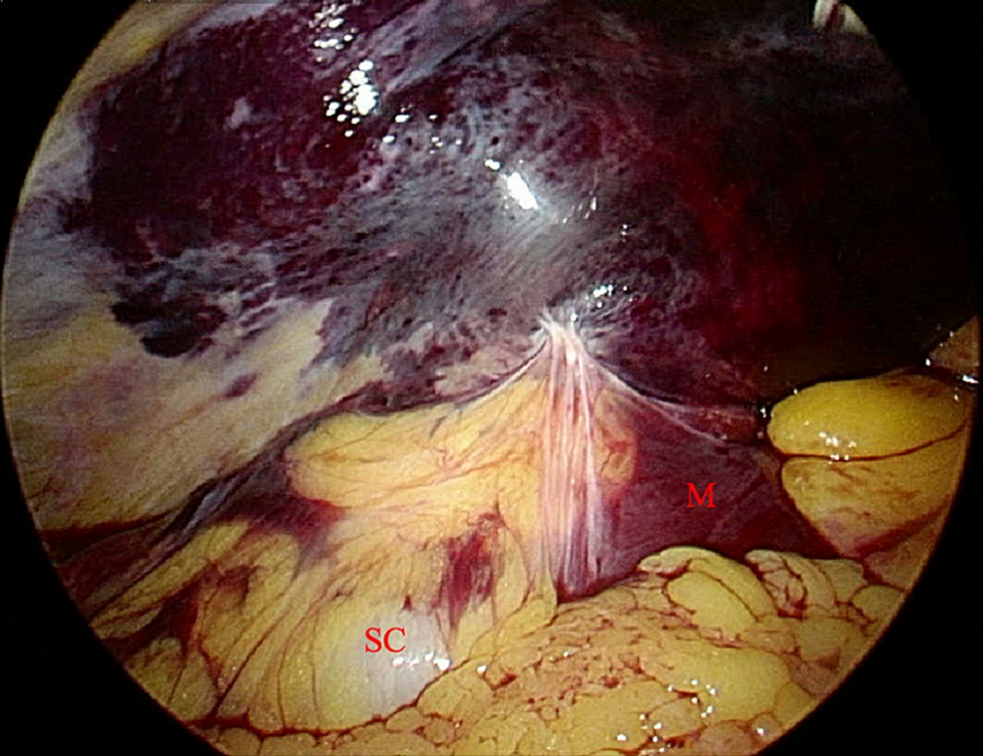
Laparoscopic image of the retroperitoneal hematoma with the camera placed in the left upper quadrant at Palmer’s point, directed at the left abdominal sidewall. SC, sigmoid colon; M, mesentery

A 45-year-old female G4P3013 with a history of heavy menstrual bleeding failed endometrial ablation, mild stress urinary incontinence, and posterior and anterior vaginal prolapse was scheduled to undergo elective total vaginal hysterectomy, bilateral salpingectomy, bilateral uterosacral ligament suspension, anterior colporrhaphy and posterior colpoperineorrhaphy, and cystoscopy. The procedure began with posterior colpotomy and suture-ligation of bilateral uterosacral ligaments. The hysterectomy involved use of a Heaney clamp technique to grasp, cut, and suture-ligate both uterine arteries. The broad ligament was secured with Heaney fixation stitches until reaching the utero-ovarian-tubo-round ligament pedicle, which was bilaterally secured with Heaney clamps. The uterus was removed, and the pedicles at this point were noted to be hemostatic. Bleeding was noted at the vaginal cuff and the left uterine artery at the site of Heaney fixation ligation sutures. The bleeding vessel was grasped with a Heaney clamp, and a subsequent Heaney fixation suture successfully established hemostasis. The vaginal cuff was irrigated, and evaluation determined that there was no bleeding from the left pelvic sidewall. Cystoscopy is commonly performed after hysterectomies to assess for lower urinary tract damage; early cystoscopy with fluorescein was performed to evaluate for possible ureteral damage caused by the extra uterine artery hemostasis stitch. Cystoscopy revealed brisk efflux of urine from bilateral ureteral orifices, and direct visualization of the pelvic cavity through the colpotomy revealed no intraperitoneal egress of urine from the ureters. The procedure continued with anterior colporrhaphy, where the anterior colpotomy was grasped by Allis clamps and an incision was created in the midline from the mid-urethra to the colpotomy. The vagina was dissected away from the underlying fascia. A Kelly-Kennedy plication was performed sub-urethrally to address the patient’s mild stress urinary incontinence. A classic three-layer plication of the redundant bladder was performed. Excess vaginal mucosa was removed, and the vaginal epithelium was closed ensuring no dead space was left between the fascia and epithelium. At this point, the patient became hemodynamically unstable; she was hypotensive, exhibited pallor, and required IV support with albumin and crystalloid. There was no bleeding noted from the vagina despite clinical evidence of hypoperfusion. Point of care hemoglobin (Hgb) testing confirmed that she was severely anemic with Hgb of 5.3 g/dL, a four-point drop from her pre-operative Hgb of 9.1 g/dL. An arterial line was then placed as the patient remained hypotensive. The decision was made to close the vaginal incision, abort the scheduled posterior colpoperineorrhaphy, and perform a diagnostic laparoscopy instead to evaluate for occult bleeding. The differential for hemorrhage included loosened vascular pedicles of the uterine arteries, bladder perforation, ureteral injury, or damage to the vaginal plexus or inferior vesical vessels. The previous cystoscopy eliminated the suspicion of bladder or ureteral damage, and the colpotomy was hemostatic at its closure, making vaginal plexus or vesical vessels an unlikely source. Therefore, the decision to perform exploratory laparoscopy was made to evaluate for intra-abdominal and RP hematoma. This revealed minimal free blood in the intraperitoneal cavity, and a large, RP and left lateral pelvic sidewall hematoma beginning at the vaginal cuff and extending to the left abdominal sidewall and mesentery, lateral to the reflection of the sigmoid colon (Figures 1-2).

**Figure 3:**
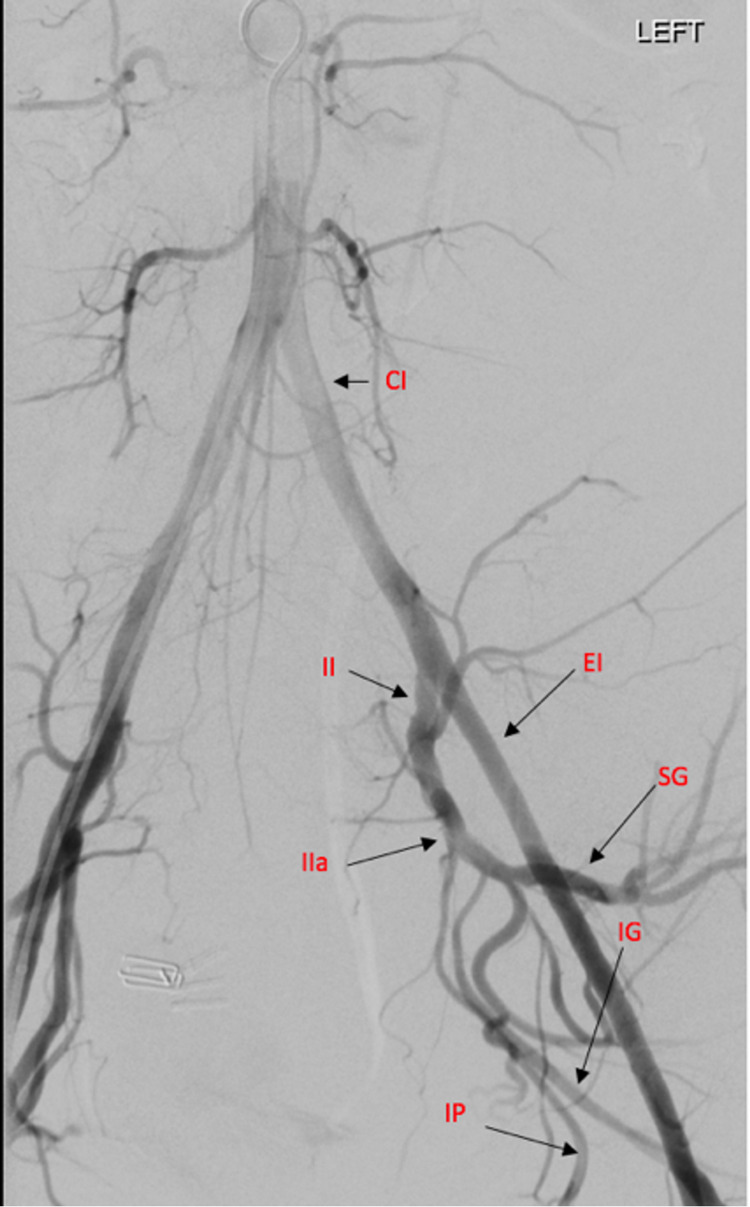
Aortogram demonstrates no extravasation from left internal iliac branches. The left uterine artery is not definitively identified, likely due to a combination of intraoperative ligation and spasm. More selective angiograms from the left internal iliac artery (not shown) also do not demonstrate extravasation. CI, common iliac artery; II, internal iliac artery; EI, external iliac artery;IIa, anterior division of internal iliac artery; SG, superior gluteal artery; IG, inferior gluteal artery; IP, internal pudendal artery

Acute care surgery was intraoperatively consulted for evaluation and management of the hematoma. The decision was made to transfer the patient to the hybrid operating room. Intervention Radiology (IR) was consulted intraoperatively to evaluate for active bleeding that could be potentially controlled endovascularly. During this time, the pneumoperitoneum was reduced to minimize potential tamponade effects, and the hematoma did not expand. The anesthesia team continued resuscitative measures and blood transfusion. After resuscitation efforts by anesthesia, the patient’s hemodynamics stabilized, and Hgb was 8.5 g/dL. Angiography revealed that the patient had neither extravasation from the left internal iliac artery nor its branches (Figure 3).

**Figure 4:**
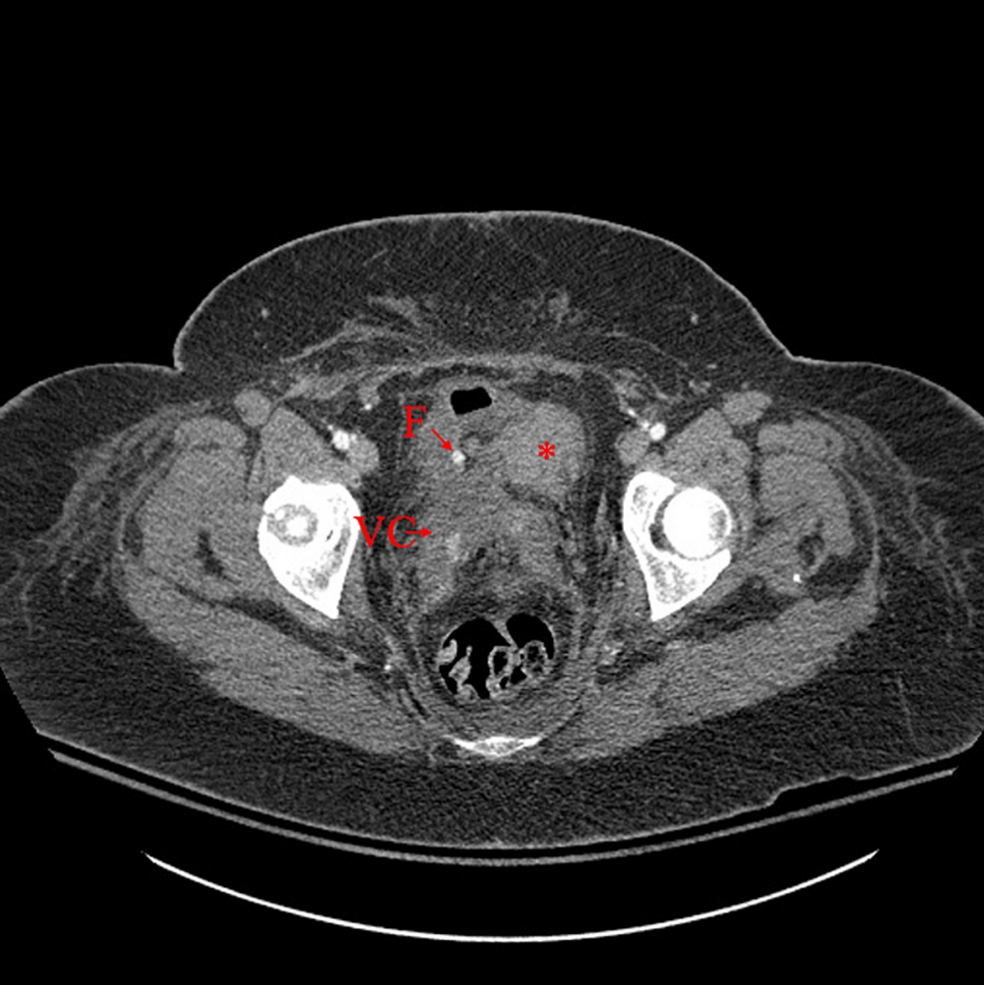
Axial CT demonstrating caudal margin of the retroperitoneal hematoma alongside the vaginal cuff anteriorly, causing mass effect on the bladder to the patient’s midline. VC, vaginal cuff; F, Foley catheter; *, retroperitoneal hematoma

**Figure 5:**
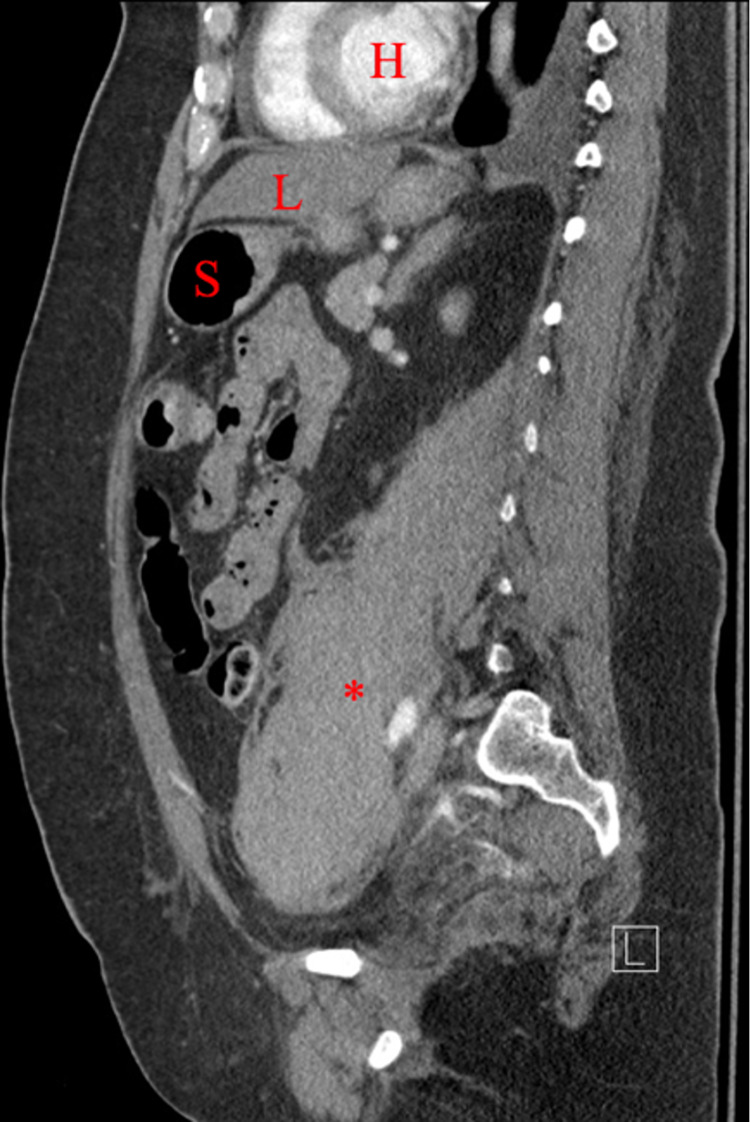
Sagittal CT demonstrating the retroperitoneal hematoma at the level of the sacrum, medial to the sacroiliac joint. H, heart; L, liver; S, stomach; *, retroperitoneal hematoma

**Figure 6:**
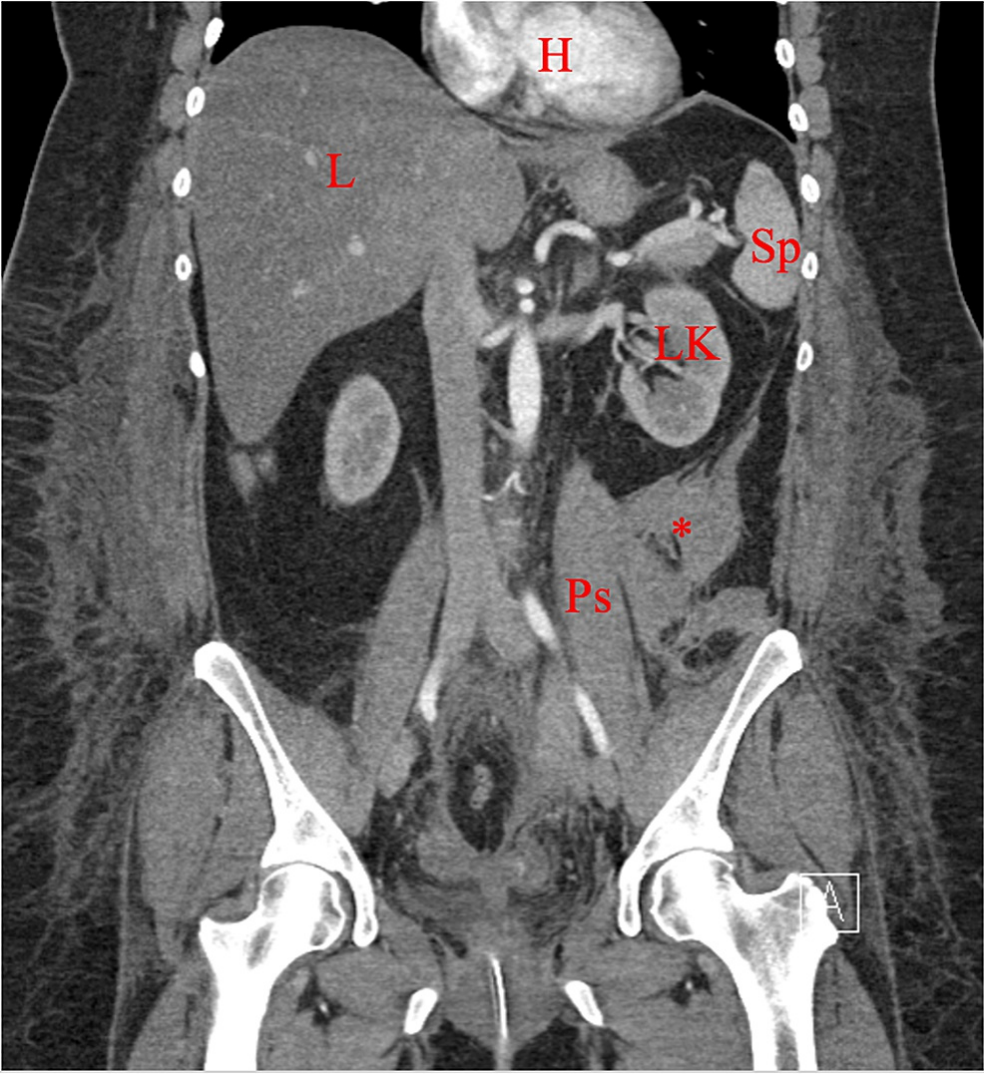
Coronal CT demonstrating retroperitoneal hematoma at the level of the femoroacetabular joint. H, heart;L, liver; Sp, spleen; LK, left kidney; *, retroperitoneal hematoma; Ps, psoas muscle

The left gonadal artery was not clearly visualized during angiography. A discussion was held between the interventional radiologist and the surgeon as to whether empiric embolization of the anterior division of the left internal iliac artery should be performed with an injectable absorbable gelatin sponge as a temporary hemostatic agent. The team decided against embolization because the patient’s condition had stabilized, and the risks of embolization, including difficulty with post-operative healing and gelatin migration, outweighed the benefits. No further interventions or explorations of the hematoma were pursued. After the procedure, the patient was transferred to the ICU for continued resuscitation. Postoperative CT was obtained to have a baseline evaluation of the hematoma size. A hematoma in the left adnexa extending cephalad into the infrarenal and left posterior pararenal spaces, measuring about 2 cm cephalocaudally, 8 cm anteroposterior, and 6 cm transverse (Figures 4-6) was revealed.

The patient continued to recover uneventfully until four days post-procedure, at which point she became hypotensive and tachycardic at blood pressure of 94/61 mmHg and pulse 107, with nausea and dizziness. Hgb was 6.4 g/dL and decreased further to 4.8 g/dL, so the patient received two units of packet red blood cell (PRBC) which improved her status; the next morning, her Hgb was 9.5 g/dL. This symptomatic anemia was re-evaluated with repeat abdominal CT, but no expansion of the RP hematoma was found. The patient was followed by both the acute care surgery services and gynecology services throughout the remainder of her hospitalization, and experienced no further adverse events. On outpatient follow-up, the patient has been stable, and therefore repeat abdominal CT was not medically indicated.

## Discussion

The etiology of the patient's RP hematoma cannot be definitively determined, given that the angiogram demonstrated an absence of extravasation from the left internal iliac branches. It is possible that the left gonadal artery or left uterine artery was the source of hemorrhage, as these vessels were not clearly identified by angiogram. It is more likely that the left uterine artery is the etiology, given its initial instability, with hemostasis established by Heaney fixation suture. The ambiguity of the source of the RP hematoma is a reflection of the possible hazard of such a surgical complication. An RP hematoma is an event that requires a prompt and efficient response; the inability to identify the responsible vessel can delay necessary treatment. Therefore, it is important to include RP hematoma on the differential for hemodynamic instability during gynecologic surgery, with the goal of initiating timely medical care.

Some of the more common early complications of hysterectomies include intraoperative and postoperative hemorrhage, hemorrhage requiring blood transfusion, infection, unexplained fever, pneumonia, and injury to adjacent organ. Diagnosis of intraoperative hemorrhage can be subjective, but generally, diagnosis is based on blood loss greater than 1000 mL, a need for blood transfusion given intraoperatively, and/or a decrease in Hgb greater than 3-5 g/dL. Post-operative hemorrhage often requires operative intervention, including direct pressure or vaginal packing, suturing in the examination room, or radiologic occlusion of the hypogastric artery [2]. 

Retroperitoneal hematoma is defined as bleeding into the RP space. It can be initially difficult to diagnose and is a cause of significant morbidity and mortality. RP hematomas can be traumatic, iatrogenic, or spontaneous in nature. Iatrogenic causes are usually associated with percutaneous interventions or endovascular procedures. They are also characterized by location divided into three zones. Zone I is the central-medial zone, which is anatomically defined between the two psoas muscles and contains midline structures such as the abdominal aorta, pancreas, duodenum, and inferior vena cava. Zone II is the perirenal zone and is lateral to the psoas muscles. This region contains portions of the colon, ureters, and kidneys. Zone III hematomas, as seen in our patient and commonly associated with gynecological procedures, represent injuries to structures that include the bladder and many vascular structures such as the presacral veins [2]. The gold-standard diagnosis for RP hematoma is a contrast-enhanced CT. The management of RP hematomas is dependent on the type of hematoma and the clinical stability of the patient. Management options range from clinical observation to endovascular embolization to surgical exploration. 

Zone III RP hematomas are less amenable to surgical management [3]. Sunga et al. reported that 24.7% of patients with non-traumatic RP hematomas underwent embolization, while 6.7% underwent surgical procedure [4]. Warren et al. described that in a study of patients with non-traumatic spontaneous RP hematomas, the need for intervention by the interventional radiologist was only 17% of 99 patients, while the need for blood product transfusion was 80%, and admission to the ICU was 70%. However, most hematomas were found to be self-limiting and rarely require surgical intervention [5]. Internal iliac artery ligation (IIAL) is sometimes used in obstetrical practice for the emergent treatment of postpartum hemorrhage [6], though it is generally underutilized in obstetric and gynecologic practice [7]. Kaya et al. reported that in a study of 26 women with postpartum RP hematoma, of 12 who underwent IIAL, 83% had successful control of postpartum hemorrhage [8]. Despite the utility of surgical intervention for postpartum hemorrhage, it may not always provide the same benefit in the context of complications during gynecologic surgery. The current case report presents an example of a gynecologic patient who would not have benefited from surgical intervention and did not require treatment beyond diagnosis, angiographic assessment, observation, and resuscitation with blood transfusion.

The RP hematoma is a rare complication of total vaginal hysterectomy. There is only one previous case report by Lev-Gur et al. that describes a vaginal hysterectomy that resulted in a pararenal hematoma; however, this study was not able to demonstrate the hematoma expanding beyond the vaginal cuff due to limitation by ultrasonography, and their lack of utilization of CT [9]. Other cases of RP hematomas secondary to gynecologic procedures include abscess formation following the laparoscopic staging of endometrial cancer [10], and pudendal block for vaginal delivery [11], both of which required non-invasive treatment with antibiotics. Zorrilla et al. reported a case of spontaneous rupture of an ovarian artery pseudoaneurysm that required embolization of the right ovarian artery [12]. These cases demonstrate the range of severity and intervention required on a case-to-case basis for RP hematoma and the potentially unpredictable nature of such a complication.

## Conclusions

Our case describes an iatrogenic RP hematoma secondary to vaginal hysterectomy, a complication that has not been widely reported or described in the literature. Previous cases, and our case report, have demonstrated that stability, treatment, and outcome of the RP hematoma can vary between patients. The RP hematoma as a surgical complication can range in severity from benign and self-limiting to potentially devastating consequences; therefore, intervention can range from supportive and non-invasive to requiring embolization for active hemorrhage.

While IIAL has been effective for the treatment of postpartum hemorrhage, there is less information available regarding the frequency, treatment, and outcome of RP hematoma in gynecologic procedures. Future direction for the literature in this field may involve analysis of incidence and risk of RP hematoma as a complication of hysterectomy, as well as the efficacy of the intervention used and outcome in such cases. Currently, we know that it is imperative to recognize the signs of hemodynamic instability during gynecologic surgery and to consider RP hematoma in the differential diagnosis. In the case of an unstable and rapidly expanding hematoma, it is important to identify the source, as a multidisciplinary approach to management can help prevent further complications.
